# Medical Specialist Care Utilization Prior to the Explantation of Cosmetic Silicone Breast Implants: A Nationwide Retrospective Data Linkage Study

**DOI:** 10.1007/s00266-024-04047-5

**Published:** 2024-05-02

**Authors:** Annemiek S. Lieffering, Lotte Ramerman, Robert A. Verheij, Hinne A. Rakhorst, Marc A. M. Mureau, René R. W. J. van der Hulst, Juliëtte E. Hommes

**Affiliations:** 1 Nivel, Otterstraat 118-124, 3513 CR Utrecht, The Netherlands; 2 Department of Plastic, Reconstructive and Hand Surgery, Medical Spectrum Twente, Enschede, The Netherlands; 3 Department of Plastic and Reconstructive Surgery, Erasmus MC Cancer Institute, University Medical Center Rotterdam, Rotterdam, The Netherlands; 4 Department of Plastic, Reconstructive and Hand Surgery, and GROW School for Oncology and Reproduction, Maastricht University Medical Center, Maastricht, The Netherlands; 5 Department of Plastic Surgery, Isala, Zwolle, The Netherlands

**Keywords:** Breast implants, Retrospective studies, Explantation, Breast augmentation, Medical specialist care, Healthcare utilization

## Abstract

**Background:**

Explantation is the proposed treatment for breast implant illness (BII). Little is known about which medical specialists are visited and what diagnoses are made before explantation is provided as the treatment.

**Objectives:**

This study investigated medical specialist care utilization in women with cosmetic breast implants who underwent explantation compared to women who chose breast implant replacement surgery and to women without breast implants.

**Methods:**

Retrospective cohort study using data linkage with the Dutch Breast Implant Registry and the Dutch health insurance claims database. Visits to medical specialists were examined over the 3 years before explantation. A total of 832 explantation patients were matched and compared to 1463 breast implant replacement patients and 1664 women without breast implants.

**Results:**

Explantation patients were more likely to have visited > 5 different medical specialties compared to both replacement patients (12.3% vs. 5.7%; *p* < 0.001) and women without breast implants (12.3% vs. 3.7%; *p* < 0.001). Among explantation patients, women who underwent explantation because of BII were more likely to have visited > 5 different medical specialties compared to women who underwent explantation because of other reasons (25.0% vs. 11.0%; *p* < 0.001).

**Conclusions:**

Women who underwent explantation of breast implants had higher utilization of medical specialist care in the years before explantation compared to women who underwent breast implant replacement surgery and women without breast implants. Medical specialist care use was especially high among women for whom BII was the registered reason for explantation. These findings suggest further research is needed into the link between BII and the use of medical specialist care.

**Level of Evidence III:**

This journal requires that authors assign a level of evidence to each article. For a full description of these Evidence-Based Medicine ratings, please refer to the Table of Contents or the online Instructions to Authors www.springer.com/00266.

**Supplementary Information:**

The online version contains supplementary material available at 10.1007/s00266-024-04047-5.

## Introduction

There is increasing recognition of breast implant illness (BII), a constellation of non-specific health symptoms that may arise after breast implantation [1]. The majority of women report an improvement in these symptoms after their breast implants are removed [2–13]. Explantation has therefore been proposed as the main treatment option for BII [7].

Yet, data on explantation surgery show that BII is an uncommon reason for explantation in comparison with local complications such as capsular contracture and implant rupture [14]. However, these surgery data are registered by plastic surgeons and they may have difficulty in recognizing BII since there are no established diagnostic criteria. This also means that it is hard to rule out BII as a diagnosis. It is likely that the number of explantation procedures with BII as the listed reason does not accurately reflect all of the women who experienced BII symptoms and underwent implant removal as a result. Examining the health of all women undergoing explantation, not just those for whom BII is the registered reason, is essential for acquiring a more comprehensive understanding of BII.

Evaluating the women’s use of medical services is one approach to ascertain their state of health. In light of the limited understanding surrounding BII, women may find themselves caught in a frustrating cycle of seeking clarity through consultations with numerous medical specialists [15, 16]. Little is known about the extent to which women undergoing explantation seek care and the specific medical specialists they consult prior to opting for explantation. The aim of this study, therefore, was to examine women’s use of medical specialist care prior to explantation and compare it to control groups of women who chose breast implant replacement surgery and women without breast implants. We hypothesized that women who undergo explantation receive more care from a broader range of medical specialists in the years prior to explantation, compared to these control groups. Secondly, we aimed to examine differences in medical specialist care utilization between subgroups of explantation patients who undergo explantation because of BII and women who undergo explantation for other registered reasons.

## Materials and Methods

### Data Sources

For this retrospective cohort study, multiple data sources were used and linked at the patient level. Data on women who underwent explantation or replacement surgery between 2016 and 2019 were provided by the Dutch Breast Implant Registry (DBIR). Since April 2015, this population-based registry has kept track of all breast implant operations (implantation/replacement/explantation) in the Netherlands [17, 18]. In the Netherlands, breast implant surgery is exclusively performed by plastic surgeons, who are obliged to record these interventions in the DBIR.

Data on medical specialist care utilization for the years 2013 to 2019 were obtained from the Dutch health insurance claims database (available through Statistics Netherlands and Vektis). This includes the expenditure on medical care for 99% of the Dutch population, who are covered by the basic national health insurance scheme [19]. All care activities provided by medical specialists are registered, including consultations, surgery, diagnostic procedures, and medication. Specialist care takes place in hospitals, independent treatment centers, dedicated institutions for specific diseases and rehabilitation, dialysis, and audiological and radiotherapy centers. In addition, Statistics Netherlands provided individual-level data on demographic and socioeconomic characteristics of the entire Dutch population.

### Study Population

The study population comprised three groups: an explantation group and two control groups. For the explantation group, women were selected who underwent unilateral or bilateral explantation surgery for a permanent implant between January 2016 and December 2019. The control groups consisted of women without breast implants (non-recipients) and women who underwent unilateral or bilateral replacement surgery between January 2016 and December 2019, in which a permanent implant was replaced with another permanent implant (replacement group). Women were identified as non-recipients if there was no record of breast implant surgery in either the DBIR or the claims database, and they were selected from the Statistics Netherlands database.

Inclusion criteria for all three groups were age ≥ 18 years, resident in the Netherlands for the entire study period (2013–2019), and no history of breast cancer according to the DBIR and claims data. Women were only eligible for the explantation and replacement groups if their breast implantation procedure had taken place more than 3 years prior to the explantation or replacement surgery. Women who had their breast implants explanted or replaced due to hematoma, skin necrosis, wound infection, confirmed breast implant-associated anaplastic large cell lymphoma, or recall reasons were excluded.

Each woman who underwent explantation was pair-matched with two replacement patients and two non-recipients (2:1 control to case ratio) for age (within 5-year intervals), and municipality code (for non-recipients only). A matching ratio of 1:1 was accepted if only one eligible control was found.

For the explantation and replacement groups, the date of the explantation or replacement procedure was considered the index date. Non-recipients received the same index date as the matched explantation patient. The study period comprised the 3 years prior to the index date.

### Medical Specialist Care Utilization

The primary outcome was visits to medical specialists in the 3 years before the index date. The secondary outcome was the registered diagnoses prompting the visits to the medical specialist. A woman was considered to have visited a specialist if at least one care activity was registered with the medical specialty in the claims data. All medical specialties that provide secondary and tertiary care in the Dutch healthcare system were included, except for sports medicine and pediatrics. General practitioners’ primary care services were excluded.

The explantation and replacement procedure itself, including any preliminary consultations with the plastic surgeon, were not included in determining medical specialist care utilization by explantation and replacement patients.

### Patient Characteristics

Patient characteristics provided by Statistics Netherlands consisted of age, migration background [20], degree of urbanization of place of residence [21], household composition, income level, and level of education, defined as the highest educational qualification obtained (low, medium, high) [22]. The standardized household disposable income was used to determine the income level, with the categories’ cut-off values based on the standardized income of the entire Dutch population. The values low, medium, and high, respectively, were assigned to the lowest 40%, middle 40%, and top 20%. Explantation and replacement patient characteristics provided by the DBIR were determined at the date of surgery and included body mass index (BMI), smoking status, American Society of Anesthesiologists (ASA) physical status classification [23], reason for explantation/replacement surgery, and implant characteristics. BMI was categorized as underweight (< 18.5 kg/m^2^), normal weight (18.5–24.9 kg/m^2^), overweight (25.0–29.9 kg/m^2^), and obese (≥ 30 kg/m^2^) [24]. BII was one of the possible reasons for explantation or replacement surgery, which was registered by the surgeon based on his/her assessment of the patient’s non-specific health symptoms or if the patient reported suffering from health problems due to her breast implants.

### Statistical Analysis

Descriptive statistics were calculated for all patient characteristics. Differences in visits to medical specialists between explantation patients and replacement patients, and between explantation patients and non-recipients, were tested using Pearson’s chi-square tests. Subgroup analyses were performed with the explantation group to examine differences in visits to medical specialists based on the registered reason for explantation (BII, implant rupture, breast pain), implant age (< 15 years, ≥ 15 years), and patient age at explantation (< 65 years, ≥ 65 years).

Furthermore, the frequency of occurrence of the 15 most common diagnoses among explantation patients was compared to the occurrence of those diagnoses among the control groups, using Pearson’s chi-square tests. All tests were carried out using STATA 16.1 (Stata Corporation, College Station, TX, USA). A two-sided *p* value of < 0.05 was considered significant.

## Results

### Patient Characteristics

In total, 832 explantation patients, 1463 replacement patients, and 1664 non-recipients were included (Figure, Supplemental Digital Content 1). The median time from implantation up until explantation or replacement was 15.0 years (IQR explantation 10.0–21.0; IQR replacement 10.0–20.0; Table 1). The most common reason for explantation and replacement surgery was capsular contracture. BII was registered in 9.1% (76) of explantation patients, and the implants were ruptured in 14 of these cases (18.4% of women with BII). Compared to replacement patients, explantation patients were older, were more likely to be overweight, and had worse preoperative health according to the ASA classification. Breast implant characteristics are described in Supplemental Digital Content 2.

**Table 1:** Characteristics of the study population

Demographics at index date	Explantation patients (*n* = 832)	Replacement patients (*n* = 1463)	Non-recipients (*n* = 1664)
*Characteristics of all 3 groups*			
Age in years, mean ± SD	54.0 ± 12.7	51.6 ± 12.0	54.0 ± 12.7
Migration background			
No	80.8% (672)	82.2% (1203)	78.3% (1302)
Yes	19.2% (160)	17.8% (260)	21.8% (362)
Degree of urbanization of place of residence			
Extremely urbanized	23.9 (199)	26.2 (383)	23.9 (398)
Strongly urbanized	31.6 (263)	32.5 (475))	31.6 (526)
Moderately urbanized	19.0 (158)	19.3 (283)	19.0 (316)
Hardly-not urbanized	25.5 (212)	22.0 (322)	25.5 (424)
Household composition			
Single parent	9.6 (80)	14.2 (207)	9.0 (150)
Couple with children	32.7 (272)	36.2 (529)	37.2 (619)
Couple without children	36.4 (303)	31.0 (454)	34.8 (579)
Other	21.3 (177)	18.7 (273)	19.0 (316)
Income level			
Low	32.1 (267)	30.7 (449)	29.7 (494)
Medium	41.4 (344)	37.0 (541)	45.8 (762)
High	26.6 (221)	32.3 (473)	24.5 (408)
Educational level			
Low	15.8 (131)	19.1 (279)	13.5 (225)
Medium	22.4 (186)	24.7 (362)	22.1 (368)
High	22.8 (190)	15.5 (227)	20.9 (348)
Unknown	39.1 (325)	40.7 (595)	43.5 (723)
*Characteristics of cosmetic breast implant surgery groups*			
Time to explantation/replacement (median, IQR) years	15.0 (10.0–21.0)	15.0 (10.0–20.0)	N/A
Smoking status			N/A
Non-smoker	73.2 (609)	66.4 (972)	
Smoker	12.9 (107)	17.4 (255)	
Unknown	13.9 (116)	16.1 (236)	
BMI			N/A
< 18.5	2.8 (23)	3.1 (45)	
18.5–25	61.5 (512)	67.7 (990)	
25–30	24.4 (203)	17.8 (261)	
≥ 30	8.8 (73)	4.8 (70)	
Unknown	2.5 (21)	6.6 (97)	
ASA classification			N/A
I	53.9 (448)	68.8 (1006)	
II	37.7 (314)	27.3 (400)	
III-V	7.8 (65)	3.5 (51)	
Unknown	0.6 (5)	0.4 (6)	
Reason for explantation/replacement^a^			N/A
*Breast Implant Illness (BII), total*	9.1 (76)	< 10^b^	
No other reason than BII	6.3 (52)	NA	
With implant rupture	1.7 (14)	NA	
With breast pain	1.9 (16)	NA	
Other reasons for explantation/replacement			
Capsular contracture	41.8 (348)	47.4 (694)	
Breast pain	24.0 (200)	16.6 (240)	
Implant rupture	33.9 (282)	33.2 (485)	
Asymmetry	6.4 (53)	15.0 (219)	
Silicone extravasation	22.0 (183)	17.5 (256)	
Device malposition	3.6 (30)	11.1 (163)	
Dissatisfaction with volume	3.1 (26)	18.0 (263)	
Other local complication^c^	5.3 (44)	2.8 (41)	
Unknown	20.9 (174)	15.0 (220)	

^a^Multiple complications can be registered as reasons for explantation/replacement, thus, percentages do not add up to 100%

^b^Exact number of observations not reported due to disclosure risk guidelines

^c^Includes flap problem, skin scarring, seroma, BIA-ALCL suspicion

### Medical Specialist Visits

**Fig. 1:**
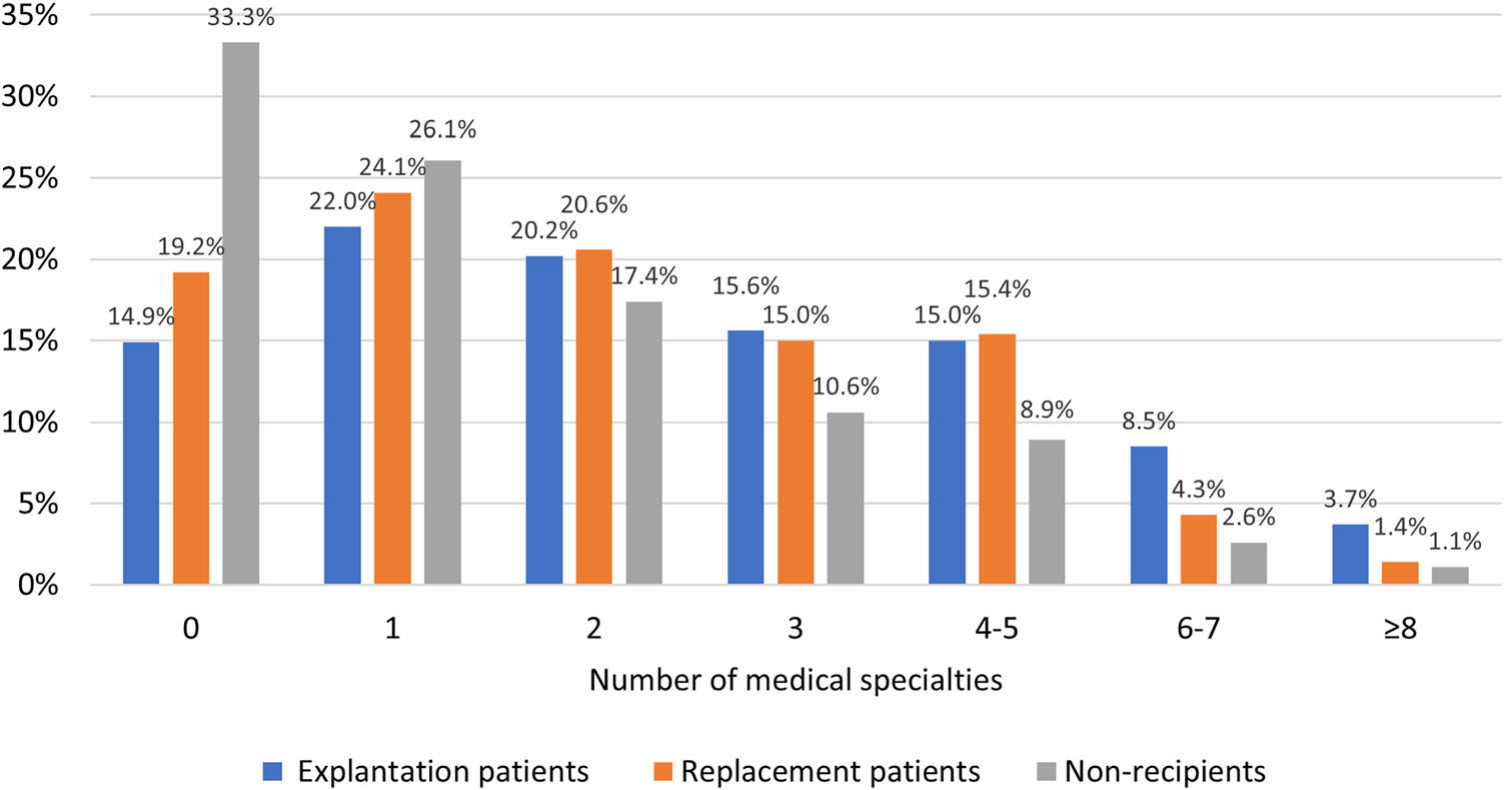
Number of medical specialties that were visited during the 3 years before the index date.

In accordance with our hypothesis, explantation patients showed higher medical specialist care use compared to both replacement patients and non-recipients (Table 2). In particular, specialists in internal medicine (20.2%), orthopedics (22.5%), neurology (20.7%), ophthalmology (19.2%), and cardiology (19.6%) were visited by a greater proportion of explantation patients compared to the control groups. Also, explantation patients were more likely to have visited more than five different medical specialties compared to both replacement patients (12.3% vs. 5.7%; *p* < 0.001) and non-recipients (12.3% vs. 3.7%; *p* < 0.001) (Table 2; Fig. 1).

**Table 2:** Number of women that visited a medical specialist during the 3 years before index date

Specialist	Explantation *n* = 832	Replacement *n* = 1463	*p* value	Non-recipients *n* = 1664	*p* value
Any medical specialist	85.1 (708)	80.8 (1182)	< 0.01	66.7 (1110)	< 0.001
> 5 different specialties	12.3 (102)	5.7 (84)	< 0.001	3.7 (62)	< 0.001
General surgery	28.4 (236)	29.9 (437)	0.45	17.1 (285)	< 0.001
Plastic surgery^a^	13.6 (113)	14.1 (206)	0.74	4.9 (81)	< 0.001
Internal medicine	20.2 (168)	16.1 (236)	0.01	12.1 (201)	< 0.001
Orthopedics	22.5 (187)	16.4 (240)	< 0.001	15.0 (249)	< 0.001
Obstetrics and gynecology	23.4 (195)	23.9 (350)	0.79	17.4 (289)	< 0.001
Neurology	20.7 (172)	16.2 (237)	< 0.01	11.8 (196)	< 0.001
Ophthalmology	19.2 (160)	14.2 (208)	< 0.01	14.3 (238)	< 0.01
Cardiology	19.6 (163)	13.2 (193)	< 0.001	10.7 (178)	< 0.001
Dermatology	22.6 (188)	20.9 (306)	0.35	15.3 (255)	< 0.001
Otolaryngology	16.0 (133)	12.3 (180)	0.01	9.2 (153)	< 0.001
Lung diseases	9.9 (82)	8.5 (124)	0.27	6.3 (104)	< 0.001
Gastroenterology	13.6 (113)	10.3 (150)	0.02	7.8 (130)	< 0.001
Rheumatology	11.4 (95)	6.7 (98)	< 0.001	4.6 (76)	< 0.001
Urology	4.8 (40)	4.2 (62)	0.52	2.3 (39)	0.001
Rehabilitation	5.2 (43)	3.2 (47)	0.02	2.8 (46)	< 0.01
Anesthesiology	5.8 (48)	4.0 (59)	0.06	2.2 (36)	< 0.001
Other^b^	5.1 (42)	5.3 (78)	0.77	3.7 (61)	0.10

*p* values calculated with *X*^2^ test

^a^Visits to plastic surgery specialists excluding the explantation and replacement procedure itself and any preliminary consultations for the procedure

^b^Psychiatry, geriatrics, allergology, neurosurgery, cardiothoracic surgery, radiology, clinical genetics, radiation therapy

### Explantation Subgroup Analyses

Women who underwent explantation because of BII were significantly more likely to have visited more than five different medical specialties compared to women who underwent explantation because of other reasons (25.0% vs. 11.0%; *p* < 0.001; Table 3). In addition, they were significantly more likely to have visited specialists in internal medicine, orthopedics, obstetrics and gynecology, cardiology, dermatology, and rheumatology compared to women with other reasons for explantation, with percentages ranging from 23.7 to 51.3% (Table 4).

**Table 3:** Subgroup analysis in explantation patients (*n* = 832)

Subgroups	Total % (*n*)	Visited >5 different specialties in the 3 years prior to explantation		
		Yes	No	*p* value
Breast implant illness				< 0.001
Yes	9.1 (76)	25.0 (19)	75.0 (57)	
No	90.9 (756)	11.0 (83)	89.0 (673)	
Breast pain				0.27
Yes	24.0 (200)	14.5 (29)	85.5 (171)	
No	76.0 (632)	11.6 (73)	88.4 (559)	
Implant rupture				0.06
Yes	33.9 (282)	9.2 (26)	90.8 (256)	
No	66.1 (550)	13.8 (76)	86.2 (474)	
Implant age				0.03
<15 years	47.5 (395)	14.9 (59)	85.1 (336)	
≥15 years	52.5 (437)	9.8 (43)	90.2 (394)	
Age at explantation				0.64
<65 years	77.3 (643)	12.0 (77)	88.0 (566)	
≥65 years	22.7 (189)	13.2 (25)	86.8 (164)	

*p* values calculated with *X*^2^ test

**Table 4:** Subgroup analysis in explantation patients (*n* = 832), stratified by breast implant illness (yes/no) as reason for explantation

Medical specialty	Breast implant illness		*p* value
	Yes (*n* = 76)	No (*n* = 756)	
Internal medicine	51.3 (39)	17.1 (129)	< 0.001
General surgery	32.9 (25)	27.9 (211)	0.36
Obstetrics and gynecology	32.9 (25)	22.5 (170)	< 0.05
Orthopedics	31.6 (24)	21.6 (163)	< 0.05
Dermatology	31.6 (24)	21.7 (164)	< 0.05
Cardiology	29.0 (22)	18.7 (141)	< 0.05
Neurology	25.0 (19)	20.2 (153)	0.33
Rheumatology	23.7 (18)	10.2 (77)	< 0.001
Ophthalmology	19.7 (15)	19.2 (145)	0.91
Otolaryngology	19.7 (15)	15.6 (118)	0.35
Gastroenterology	18.4 (14)	13.1 (99)	0.20
Plastic surgery^a^	17.1 (13)	13.2 (100)	0.35
Lung diseases	14.5 (11)	9.4 (71)	0.16
Other^b^	23.7 (18)	16.3 (123)	0.10

*p* values calculated with *X*^2^ test

^a^Visits to plastic surgery specialists excluding the explantation procedure itself and any preliminary consultations for the procedure

^b^Psychiatry, geriatrics, allergology, neurosurgery, cardiothoracic surgery, radiology, clinical genetics, urology, rehabilitation, anesthesiology, radiation therapy

Also, women who had had their implants for less than 15 years at the time of explantation were significantly more likely to have visited more than five different medical specialties compared to women who had their implants for more than 15 years until explantation (14.9% vs. 9.8%; *p* = 0.03; Table 3). There were no significant differences in the number of women who visited more than five medical specialties after stratification by breast pain and implant rupture as the reason for explantation, and patient’s age (Table 3).

### Diagnoses

The most common diagnosis in the 3 years preceding explantation was mastopathy (11.9%; Table 5). A similar number of replacement patients were diagnosed with mastopathy as well (12.1%), while among non-recipients, fewer women were diagnosed with mastopathy (3.1%). Other common diagnoses among explantation patients included thoracic complaints (7.2%), analysis of systemic disorder without diagnosis (3.9%), fatigue without diagnosis (2.9%), and sinusitis (2.9%). Lower frequencies of these diagnoses were seen in both replacement patients and non-recipients (*p* < 0.05).

**Table 5:** Most common diagnoses among explantation patients during the 3 years prior to explantation, compared to frequencies in both control groups

Top 15 diagnoses explantation	Explantation patients	Replacement patients	*p* value	Non-recipients	*p* value
	% (*n*) of total population (*n* = 832)	% (*n*) of total population (*n* = 1463)		% (*n*) of total population (*n* = 1664)	
Benign neoplasm mamma/mastopathy	11.9 (99)	12.1 (177)	0.89	3.1 (51)	< 0.001
Thoracic complaints e.c.i.	7.2 (60)	4.4 (64)	< 0.01	3.8 (63)	< 0.001
Menstruation disorder	5.1 (42)	4.9 (72)	0.89	3.6 (59)	0.07
Malignant skin disorder	4.1 (34)	3.2 (47)	0.28	2.8 (47)	0.09
Analysis systemic disorder without diagnosis	3.9 (32)	< 10	< 0.001	< 10	< 0.001
Cataract	3.5 (29)	2.1 (31)	0.049	2.9 (49)	0.46
Cervical abnormality	3.4 (28)	4.8 (70)	0.11	2.6 (44)	0.31
No evidence of cardiac abnormalities	3.4 (28)	2.4 (35)	0.17	1.1 (19)	< 0.001
Premalignant skin disorder	3.0 (25)	3.2 (47)	0.78	2.3 (38)	0.28
Sensorineural hearing impairment	3.0 (25)	2.1 (30)	0.15	1.9 (32)	0.09
Sinusitis	2.9 (24)	1.4 (20)	0.01	1.0 (17)	0.001
Gonarthrosis	2.9 (24)	2.1 (30)	0.21	3.6 (60)	0.35
Analysis general malaise/fatigue without diagnosis	2.9 (24)	1.0 (14)	0.001	0.8 (13)	< 0.001
Arthralgia and/or myalgia	2.9 (24)	1.9 (28)	0.13	1.0 (17)	0.001
Incontinence	2.8 (23)	1.6 (23)	0.05	1.5 (25)	0.03

*p* values calculated with *X*^2^ test

## Discussion

In this retrospective cohort study, our objective was to examine the utilization of medical specialist care in women who underwent explantation surgery. Explantation is the proposed main treatment for BII, and by comparing the medical specialist care received by these women with the medical specialist care received by control groups of women who underwent replacement surgery and women without breast implants, we aimed to gain insight into the health status of these women prior to explantation. Given the absence of established diagnostic criteria for BII, our investigation encompassed all women undergoing explantation, not just those with BII as the registered reason. We found that women who underwent explantation of their cosmetic breast implant(s) had significantly higher levels of medical specialist care use in the preceding 3-year period than women who underwent breast implant replacement surgery and women without breast implants. This medical specialist care utilization was particularly high among women in whom BII was the registered reason for explantation, especially for visits to specialists in internal medicine, orthopedics, obstetrics and gynecology, cardiology, dermatology, and rheumatology.

We believe that the results of our BII subgroup analysis suggest that medical specialist care utilization could serve as a relevant indicator of BII. A quarter of women who underwent explantation because of BII visited more than five different medical specialties before explantation. Yet, women without the label BII in the DBIR database still had significantly higher medical specialist care utilization compared to replacement patients and women without breast implants. These women may have been in poor health due to medical conditions unrelated to the implants. However, it could also indicate that there are women who underwent explantation due to BII complaints, but who were not registered as BII cases. The registration of BII in the DBIR depends on whether the plastic surgeon recognizes BII or whether the patient addresses it as the reason for explantation, which is challenging in the absence of diagnostic criteria. Thus, BII may be more prevalent than explantation surgery data indicate [14]. There is an urgent need for validated diagnostic criteria to determine the prevalence of BII, and to improve care for these women with health complaints. Our findings constitute an important signal that women who undergo explantation have a significant need for medical specialist care prior to the procedure, which warrants more research.

Previous research into healthcare utilization due to BII and other long-term complications of breast implants is lacking. In one previous study, questionnaires were used to examine healthcare use among Dutch women with breast implants who had self-reported health complaints [15]. Compared to our study, fewer women visited medical specialists; 32% reported seeing an internist for their complaints, while in our study, 51% of women who underwent explantation because of BII had visited internal medicine physicians. However, we do not know to what extent these visits were related to BII, as opposed to the prior study where women were specifically questioned about care they had received in response to their health complaints. Another reason for the higher number of visits to medical specialists in our study could be the increased media exposure and growing awareness of BII since the previous study in 2017 [25, 26].

Generally, the average latency period between implantation and the onset of BII symptoms is reported to be around 4.5 years, [2, 3, 27, 28]. Women who chose to have their breast implants removed due to health symptoms are reported as doing so after an average of 8.5 to 14.5 years [2, 4, 6, 28], which is in line with the 15-year interval we found in our study. Thus, women who undergo explantation because of BII seem to endure health complaints for a number of years before they proceed to explantation. One reason why women only have their implants removed after years of discomfort may be because neither the women nor their physicians were aware of the possible link between breast implants and their symptoms. Another reason may be the costs involved in the explantation procedure. For example, in the Netherlands, the cost of were rarely reimbursed by health insurers before 2021 if the operation was performed solely because of BII [29, 30]. Finally, explantation leads to a reduction in breast volume, a feared consequence for many women with cosmetic breast implants.

Our study showed that the use of medical specialist care in the years preceding explantation involved more than just visits to the plastic surgeon for the procedure itself. Inherently, this may imply that the total costs of care related to BII symptoms may comprise more than the costs of the explantation procedure alone. This would underscore the importance of prompt recognition of BII by medical professionals in order to start the conversation about explantation as a possible treatment option [4, 7]. Current financial reimbursement initiatives in the Netherlands are a step in the right direction to facilitate this. Since 2021, the costs of explantation have been reimbursed if an internist or clinical immunologist has determined that the symptoms only started after breast implantation, have persisted for at least 12 months, and have no other known causes.

The present study faced difficulties in assessing the characteristics of women with BII as the registered reason for explantation owing to the population group size. The use of routinely recorded data was subject to disclosure risk guidelines, constraining the publication of patient characteristic data with few observations. Nevertheless, after investigating the total population of explantation patients, notable trends emerged. Specifically, these women seemed to be older, more likely to be overweight, and to have worse preoperative health compared to the control group of replacement patients. Overweight is known to be an important determinant of health and healthcare utilization [31]. Unfortunately, BMI was recorded only on the surgery date, and long-term weight information remained unavailable, preventing a comprehensive assessment of the influence of BMI on explantation in this study. However, the percentage of overweight women among the women who underwent explantation (BMI ≥ 25: 33.2%) was still lower than the percentage in the general adult female population in the Netherlands [32]. Moreover, the higher percentage of overweight women in explantation patients compared to replacement patients may be directly related to the patient’s choice to undergo explantation. Generally, women with higher BMI often have naturally larger breasts, potentially reducing their desire to maintain breast enlargement through implants [33]. Nonetheless, the exploration of these and other patient characteristics’ impact on BII incidence merits special attention in future studies. Due to the improved reimbursement possibilities after 2020 for women with BII, we expect an increase in registration of BII among explantation patients in the coming years. This anticipated growing patient population will allow for better analysis of patient characteristics and the potential determination of who is at risk of BII.

Our study has some limitations. First, a study period of 3 years prior to explantation is relatively short considering a median time of 15 years between implantation and explantation. In addition, we were unable to examine the medical specialist care utilization after explantation due to limited availability of claims data. Current evidence on the possible treatment effects of explantation on health complaints is based exclusively on self-reported symptoms by patients. Thus, it would be particularly useful to repeat the current study with bias-free claims data when data covering several years after explantation become available.

Second, we examined medical specialist care use without selecting diagnoses or care activities, and without correcting for chronic diseases and other confounders. Therefore, no direct conclusions can be made regarding the relationship between breast implants and specific health outcomes.

## Conclusion

One in eight women with cosmetic breast implants who underwent explantation visited more than five different medical specialties in the 3 years prior to explantation. This translates to a level of medical specialist care utilization that is two to three times higher compared to women who underwent replacement of their cosmetic breast implants and women without breast implants, respectively. Medical specialist care use was especially high among women in whom BII was the registered reason for explantation. These findings underscore the substantial healthcare needs of women who eventually undergo explantation, emphasizing the importance of timely recognition of BII by healthcare providers. Future studies should investigate post-explantation healthcare utilization and prioritize the development and validation of diagnostic criteria for BII, with the aim of improving its identification within clinical practice.

## Supplementary Information

Below is the link to the electronic supplementary material.

Supplementary file1 (DOCX 27 kb)

Supplementary file2 (DOCX 13 kb)
