# Supplementary material for: Medical Specialist Care Utilization Prior to the Explantation of Cosmetic Silicone Breast Implants: A Nationwide Retrospective Data Linkage Study
Source: Aesthetic Plast Surg. 2024 May 2;48(21):4404–13. doi: 10.1007/s00266-024-04047-5 (PMC11588962; doi:10.1007/s00266-024-04047-5)
Supplement: Supplementary file 1 — Supplementary file1 (DOCX 27 kb) [file 266_2024_4047_MOESM1_ESM.docx]

Women (≥18 yrs.) who underwent explantation surgery (2016-2019)

n = 1,824

Women (≥18 yrs.) with no breast implant surgery (2015-2020)

n = 10,424,441

Excluded total n = 991

Non-resident during study period (3 years before explantation) (n = 39)

Hematoma, skin necrosis, wound infection, confirmed BIA-ALCL, recall (n = 192)

Year of implantation unknown (n = 502)

Implantation took place during study period (n = 71)

History of breast cancer (n=187)

1:2 matching

Explantation patients

n = 832

Non-recipients

n = 1664

Eligible for inclusion

n = 833

Eligible for inclusion

n = 6,143,955

Excluded total n = 4,280,486

Non-resident or deceased during study period (2013-2019) (n = 4,086,854)

History of breast cancer (n= 193,632)

Age (5 year intervals) and municipality code^a^

^a^Only applied to matching with non-recipients

Women (≥18 yrs.) who underwent replacement surgery (2016-2019)

n = 4,572

Eligible for inclusion

n = 1,923

Excluded total n = 2,649

Non-resident during study period (3 years before replacement) (n = 70)

Hematoma, skin necrosis, wound infection, confirmed BIA-ALCL, recall (n = 10)

Year of implantation unknown (n = 1818)

Implantation took place during study period (n = 246)

History of breast cancer (n=505)

Replacement patients

n = 1463

Supplemental digital content 1 – flow diagram of study population selection
