# Supplementary material for: Medical Specialist Care Utilization Prior to the Explantation of Cosmetic Silicone Breast Implants: A Nationwide Retrospective Data Linkage Study
Source: Aesthetic Plast Surg. 2024 May 2;48(21):4404–13. doi: 10.1007/s00266-024-04047-5 (PMC11588962; doi:10.1007/s00266-024-04047-5)
Supplement: Supplementary file 2 — Supplementary file2 (DOCX 13 kb) [file 266_2024_4047_MOESM2_ESM.docx]

| Supplemental digital content 2 – Breast implant characteristics of explantation patients and replacement patients | | |
| --- | --- | --- |
| Implant characteristics | Explantation patients  (n = 832) | Replacement patients  (n = 1463) |
| Texture of explanted/replaced implant(s) |  |  |
| Textured | 76.3 (635) | 76.6 (1121) |
| Smooth | 16.5 (137) | 11.2 (164) |
| Unknown | 7.2 (60) | 12.2 (178) |
| Coating of explanted/replaced implant(s) |  |  |
| Silicone | 92.0 (765) | 86.7 (1268) |
| Other | <10^a^ | 1.6 (23) |
| Unknown | >57^a^ | 11.8 (172) |
| Filling of explanted/replaced implant(s) |  |  |
| Silicone | 91.0 (757) | 83.9 (1227) |
| Other | 2.4 (20) | 4.5 (66) |
| Unknown | 6.6 (55) | 11.6 (170) |
| Shape of explanted/replaced implant(s) |  |  |
| Round | 69.4 (577) | 66.0 (965) |
| Anatomical | 23.7 (197) | 22.6 (330) |
| Unknown | 7.0 (58) | 11.5 (168) |
| ^a^ Exact number of observations not reported due to disclosure risk guidelines. | | |
